# Automated Inference of Social Anxiety From Behavior in Social Virtual Reality: Cross-Sectional Observational Study

**DOI:** 10.2196/79147

**Published:** 2026-03-11

**Authors:** Gayoung Son, Marius Rubo

**Affiliations:** 1 Cognitive Psychology, Perception and Research Methods Institute of Psychology University of Bern Bern Switzerland

**Keywords:** eye gaze, interaction behavior, psychopathology, social anxiety, social virtual reality, speaking behavior, verticality

## Abstract

**Background:**

Social anxiety often manifests through behaviors such as reduced gaze to the eyes and lower speech volume. While these markers have been examined in face-to-face interactions, large-scale assessments remain challenging. Social virtual reality (VR) offers a promising alternative by enabling naturalistic interactions in which behavior can be captured at scale, but it remains unclear if people show naturalistic behavior in such artificial environments.

**Objective:**

We examined whether behavioral and physiological markers associated with social anxiety in real-life interactions similarly emerge in dyadic social VR. We additionally examined whether these patterns overlap with patterns linked to the broader constructs of psychopathology and verticality.

**Methods:**

In this cross-sectional study, 128 participants (105 females, 22 males, 1 diverse; age 18-51 years; mean age 22.60, SD 3.57 years), recruited from a university student population, engaged in a 30-minute avatar-mediated dyadic conversation in social VR while physically located in separate rooms. We assessed gaze toward the partner’s eyes, smiling, and speaking behavior by using the VR headsets’ built-in eye trackers, face trackers, and microphones, and assessed high-frequency heart rate variability (HF-HRV) by using an electrocardiogram.

**Results:**

Relationships between traits and behavioral and physiological measures were analyzed using linear mixed-effects models (α=.05). Social anxiety was linked to reduced gaze toward the partner’s eyes while speaking (*β*=–.20, 95% CI –0.35 to –0.04; t_126_=–2.51; *P*=.01), quieter speech (*β*=–.18, 95% CI –0.35 to –0.01; t_126_=–2.12; *P*=.04), and reduced HF-HRV (*β*=–.23, 95% CI –0.39 to –0.08; t_119_=–3.00; *P*=.003). These findings were not entirely specific to social anxiety, as Pearson correlations revealed similar patterns for social anxiety and psychopathology (*r*=0.94, 95% CI 0.75-0.99; *t*_7_=7.61; *P*<.001), whereas verticality was linked to an overall opposite pattern (social anxiety: *r*=–0.92, 95% CI –0.98 to –0.65; *t*_7_=–6.14; *P*<.001; psychopathology: *r*=–0.83, 95% CI –0.96 to –0.38; *t*_7_=–3.98; *P*=.005).

**Conclusions:**

In dyadic interactions in social VR, social anxiety was associated with behavioral and physiological modulations similar to those observed in face-to-face interactions, indicating heightened social stress and submissiveness even in avatar-mediated communication. Patterns were similar for heightened psychopathology and reversed for verticality, indicating that these traits may lie on a shared social-behavioral spectrum. Extending previous research focused on face-to-face interactions or reactions toward artificial agents displayed in VR, this study is the first to provide a comprehensive account of the behavioral and physiological modulations associated with social anxiety in avatar-based human-human interactions. Since social VR setups allow researchers to assess a rich set of behavioral data as a byproduct of the setup’s core functionality, the technique opens novel possibilities to detect social stress, track therapeutic progress, or tailor interventions to individual behavior when interactions take place in VR.

## Introduction

### Overview

Social anxiety is a psychological trait deeply rooted in social functioning, shaping how individuals perceive and behave in social situations, often manifesting in distinct behavioral and physiological patterns during social interactions [1]. It is widely distributed across the population, with higher levels linked to increased emotional distress and impaired social functioning. Individuals with severe expressions of social anxiety can meet the diagnostic criteria for social anxiety disorder, with a lifetime prevalence of 13% and a 12-month prevalence of 8% among adults [2]. Even moderate, subclinical levels are common and linked to elevated comorbidity and reduced quality of life [3]. Capturing how social anxiety is behaviorally expressed in social interactions is essential for understanding its impact and identifying individuals at risk.

Research has identified a set of robust behavioral and physiological markers associated with social anxiety [4], often conceptualized as safety behaviors—coping mechanisms aimed at preventing negative social outcomes [1,5]. These include gaze avoidance [6], which has been shown to increase during speaking phases in individuals with higher social anxiety [7,8]. However, not all studies have observed this phase-specific pattern [9,10], suggesting that gaze behavior may vary depending on situational factors and may reflect context-sensitive manifestations of underlying traits.

Changes in facial expression have also been observed. In face-to-face interactions, individuals with higher social anxiety have been found to smile more [11], in some cases specifically mimicking the partner’s polite smiles [12], which has been suggested to reflect appeasement strategies aimed at avoiding negative evaluation. Overall, speaking behavior is also affected: social anxiety has been associated with the tendency to speak less [13], interrupt less often [14], speak more quietly, and pause longer between speaking turns [15], though these associations may be modulated by gender or context [16]. Together, these patterns suggest a tendency to limit social visibility and reduce opportunities for self-expression, possibly as a means of avoiding evaluation. In parallel, physiological studies consistently report elevated heart rate and reduced high-frequency heart rate variability (HF-HRV) in socially anxious individuals [17,18]—markers of reduced parasympathetic activity and emotional self-regulation [19,20].

While social anxiety has frequently been linked to specific behavioral markers, it is increasingly recognized that these expressions are not exclusive to the trait. Psychological traits often overlap, and patterns attributed to social anxiety may reflect broader constructs. As an internalizing condition, social anxiety shows behavioral similarities with other anxiety and mood syndromes (eg, increased gaze avoidance in depression [21]), but also with externalizing disorders (eg, reduced HF-HRV in substance use disorders [22]). These overlaps align with the notion of a general factor of psychopathology (*p*-factor), which reflects shared variance across mental disorders and has been proposed as a transdiagnostic dimension of psychopathology severity [23,24].

Similarly, verticality—a trait reflecting perceived dominance and social rank—has been linked to reduced social anxiety [25]. Individuals high in social anxiety have been shown to display more low-verticality behaviors such as gaze aversion and reduced speech [4,15,26,27], which are interpreted as submissive strategies for reducing ostracism [28-30]. In contrast, higher verticality is associated with dominant behaviors such as increased gaze, louder speech, and more frequent interruptions [31,32], suggesting an inverse relationship between these constructs.

Notably, verticality itself shows conceptual and empirical overlap with broader psychopathology, as internalizing profiles are associated with perceived inferiority [33], while externalizing traits align with dominance-seeking behavior [25]. To this end, this study assessed a range of psychological traits—including social anxiety, general psychopathology, and verticality—to examine both their shared behavioral expressions and trait-specific associations.

While these behavioral and physiological markers have been extensively researched, capturing them reliably in naturalistic settings remains a significant methodological challenge [34]. Traditional laboratory paradigms—such as eye-tracking studies using static images or videotaped face-to-face interactions—offer experimental control but have typically required restricted participant movement and relied on time-intensive manual coding [35]. To overcome these limitations, researchers have increasingly turned to digital phenotyping, which leverages passive data from smartphones to infer psychological traits [36]. For example, mobility and communication patterns have been shown to predict social anxiety symptom severity [37], while a recent systematic review found that smartphone sensor data can detect behavioral patterns linked to stress, anxiety, and mild depression in student populations [38].

While such findings demonstrate the growing use of mobile sensing, these approaches primarily rely on indirect proxies—such as phone use, physical activity, or ambient audio—that are often captured outside interactive contexts. Moreover, technical challenges such as inaccurate readings when phones are stowed, or poor audio quality in noisy environments, limit the reliability of such data [38].

Critically, smartphone sensing lacks access to the social interactive behavioral cues—such as gaze coordination, conversational timing, or facial expressivity—that are central to social anxiety in interpersonal contexts. For instance, reliably determining whether someone is making eye contact while speaking requires precise tracking of eye movements and speaking behavior of both partners—capabilities that remain difficult to scale during face-to-face interactions.

Social virtual reality (VR) directly addresses these limitations by combining immersive, ecologically valid interaction with high experimental control [39]. Participants engage through avatars that, in the most modern installments, mirror their gaze, facial expressions, gestures, and speech in real time, enabling naturalistic social behavior to be studied under replicable conditions [40,41]. Our previous publication based on the same dataset [41] documented natural interaction behavior at the group level (eg, more gaze to the eyes while listening compared to speaking; more gaze to the eyes at the end of a speaking turn compared to the beginning of a speaking turn; more smiling and interrupting in small-talk compared to more serious conversations; and preserved interaction patterns in anonymity). Building on that work, this study maps the links between interindividual differences in behavior, physiology, and psychological traits, providing detailed insights into expressions of clinically relevant personal characteristics.

Although recent studies have increasingly leveraged VR to examine ecologically valid expressions of behavior and physiological responses [42,43], the technology was traditionally used in contexts where participants interacted alone or with virtual agents [44]. Early work already demonstrated that interpersonal distance toward virtual agents with differential gaze behavior mirrored real-life proxemic behavior [45]. More recent research has begun to focus on human-human interaction within virtual environments [46], with studies investigating gaze avoidance [47], physiological synchrony [48], and facial expressions [49] in dyadic social VR settings, as well as speaking behavior and social coordination in larger group interactions [50]. Crucially, social VR enables the automated collection of multimodal behavioral data in ways comparable to established single-user techniques [51,52], highlighting its promise as a scalable platform for studying social interaction behavior. Our study contributes to this growing line of research by integrating behavioral and physiological measures within a single analytical framework to provide a more comprehensive account of real-time interpersonal dynamics in social VR.

### Objective

We assessed whether behavioral alterations associated with social anxiety (but also related constructs such as general psychopathology and verticality) can be observed in interactions in social VR, which allows for naturalistic social interactions while enabling automated and scalable measurement of interaction behavior. To the best of our knowledge, our study is the first to simultaneously assess multiple social behaviors and physiological signals during real-time dyadic interactions in VR. Participants engaged in a dyadic conversation while embodying virtual avatars that displayed their eye movements, facial expressions, and gestures in real time. We specifically assessed gaze, speaking, and smiling behavior, which are processed automatically as part of the social VR environment, but also heart rate and HF-HRV, a robust physiological indicator of psychological stress and anxiety. Specifically, the hypotheses are as follows:

- Hypothesis 1: We expected different self-report measures of social anxiety, as well as different measures of psychopathology and verticality, to load on a common underlying factor.
- Hypothesis 2: We expected higher social anxiety (and similarly, higher psychopathology but lower verticality) to be associated with behavioral alterations:
  Hypothesis 2.1: less gaze at the partner’s eyes while speaking and listening
  Hypothesis 2.2: heightened overall smiling behavior
  Hypothesis 2.3: reduced overall speaking time, fewer interruptions during the partner’s speech, longer pauses (ie, gaps before starting to speak after the partner finished their turn), and loudness of speech
- Hypothesis 3: We expected higher social anxiety (and similarly, higher psychopathology but lower verticality) to be associated with alterations in physiological functioning, specifically, heightened heart rate and reduced HF-HRV.

## Methods

### Participants

A total of 128 participants (105 female, 22 male, 1 diverse; aged 18-51 years; mean age 22.60, SD 3.57 years) participated in a dyadic interaction in the social VR environment. This predetermined sample size allows for an 85% chance of detecting a correlation of *r*=0.26 with α=.05 in a repeated-measures correlation involving 3 measures. Most participants (n=122, 95.31%) were undergraduate psychology students at the University of Bern, 118 with Swiss nationality, 8 with German nationality, and 14 from other European or non-European countries (multiple nationalities are possible). Exclusion criteria included neurological conditions, use of central nervous system–acting substances, and severe visual impairments requiring glasses that are incompatible with VR equipment. Within this sample, none of the individuals had more than a brief acquaintance with one another (n=5, 3.91% participants). To preserve conceptual clarity and avoid overlap between constructs, we kept measures distinct across factors. We included additional behavioral variables (ie, smiling and heart rate) and examined eye gaze separately during speaking and listening phases to offer a broader understanding of participant behavior. Data and analyses are accessible at [53].

### Ethical Considerations

This study was approved by the institutional review board of the University of Bern (reference 2023-09-01) and was preregistered [54]. Prior to giving consent, all participants were verbally informed of the research aims, procedure, the voluntary nature of participation, and the right to withdraw at any point in the study freely and without consequences. They were also informed that they might experience symptoms of VR sickness and were instructed to remove the headset and inform the experimenter if this occurred; however, no such instances were reported. All collected data were anonymized and managed securely. Participants received 2 course credits as compensation. All figures containing persons depict example scenes performed by the study authors and do not include actual participants.

### Procedure

**Figure 1:**
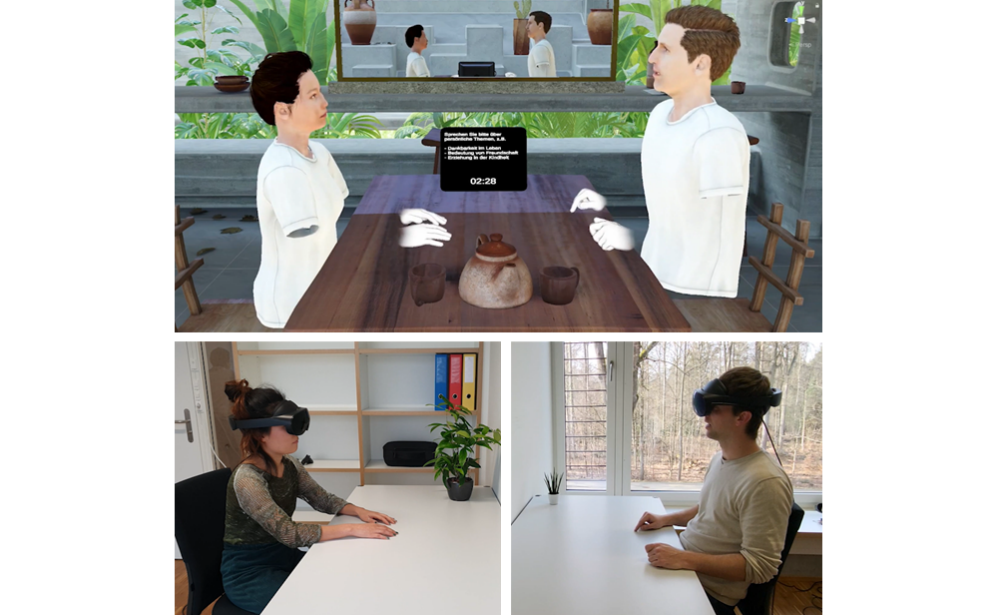
The experimental setup seen from a third-person perspective. Participants meet as avatars in virtual reality (top image) while physically located in separate rooms (bottom images). Avatars convey participants’ movements, eye gaze, and facial expressions in real time, and voice is transmitted via voice chat. The virtual environment’s position and orientation are calibrated so that the position of a virtual table matches the position of a physical table for both participants. All dyads conversed on predefined topics for a total of 30 minutes. People displayed in the images are the study authors demonstrating the experimental setup and procedure.

**Figure 2:**
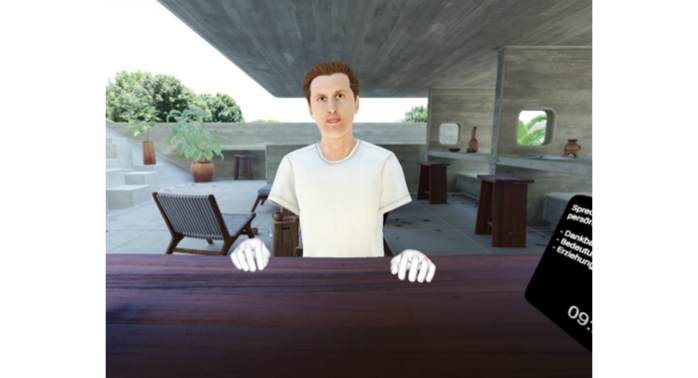
First-person perspective of a participant during the virtual reality interaction. Each participant views the scene from the perspective of their avatar’s eyes, allowing for technology-mediated direct eye contact between participants.

This cross-sectional study followed the reporting guidelines for the STROBE (Strengthening the Reporting of Observational Studies in Epidemiology) Statement for observational studies [55]. Participants were recruited through the university’s participant recruitment system between November 2023 and February 2024. Throughout the entire study (lasting approximately 1.5 hours), participants were seated in separate rooms and interacted solely through the social VR software (Figures 1 and 2), which was custom-developed in Unity 3D [56] for this study. Participants additionally arrived and departed separately in order to ensure that they did not see each other at any point. The virtual environment was displayed using Meta Quest Pro headsets [57] and ran on Windows 11 desktop laptops (Intel Core i9-13900HX CPU, 32 GB RAM, and NVIDIA GeForce RTX 4070 GPUs). Validation of study measures is reported in Son and Rubo [41]. A detailed description of the software architecture is provided in Rubo [58]. Subsequently, participants were equipped with surface electrodes to obtain an electrocardiogram throughout the entire study.

Before the interaction, all participants completed an initial set of questionnaires, which included the self-reported measures of interest. Once the questionnaires were completed, participants were introduced to the virtual environment. This involved headset calibration, a 9-point eye-tracking calibration, and adjustments to the interpupillary distance. Participants also aligned their seating height and orientation to ensure that the virtual table was positioned to correspond precisely with the physical table. Next, participants observed their avatar in a virtual mirror, allowing them to see their avatar’s full range of movements. Each avatar displayed the head and eye movement, blinking, mouth movement, facial expressions, and gestures of the participants in real time. Avatars were generated using Character Creator 4 along with the Headshot extension [59] and were configured to render only the head, upper torso, and hands. Each avatar featured extensive facial details and was individually adjusted by a research assistant for eye color, facial shape, hair, and body size (for more details on avatar creation, see Multimedia Appendix 1 [41,59-66]).

In addition, half of the dyads interacted while embodying self-similar avatars generated from participants’ portrait photos, and the other half interacted while embodying gender-matching avatars created using generic images of nonexisting individuals from a random pool of 6 male and 6 female avatars. As detailed in a separate report [41], this condition investigated the effects of avatar appearance on social interactions. Since no behavioral differences were identified, and participants furthermore reported no differences in satisfaction with their avatars or in the attractiveness of their avatars, conditions were aggregated in this study.

The experimenter then refined the avatar’s size, head orientation, and eye-tracking calibration based on participant feedback. Once the setup was complete, participants were instructed that the study would include three 10-minute conversations on predefined topics (small talk, personal talk, and opinion talk; Multimedia Appendix 1) presented in randomized order. Participants were asked to remain on topic for the duration of the task. The experimenter then exited the room, and participants were connected to their partners within the VR environment. During the interaction, participants were guided by prerecorded computerized voices, and each discussion prompt and the remaining time were displayed on a virtual tablet. After the interaction, they completed control questions on the quality of the interaction (eg, VR sickness symptoms or satisfaction with the avatar), which are described in a separate report [41]. Analyses reported here refer to interactions as a whole.

### Self-Report Measures

#### Social Interaction Anxiety

Social interaction anxiety symptoms were examined using the Social Interaction Anxiety Scale (SIAS [67]; German version [68]). The SIAS assesses anxiety in social interactions in 20 items, rated on a 5-point Likert scale (0=not at all, 4=extremely). Internal consistency measured with Cronbach α was 0.91.

#### Fear of Positive and Negative Evaluation

Fear of positive and negative evaluation is a core symptom of social anxiety, consisting of fears of both negative and positive judgment from others [69]. Fear of positive evaluation was assessed using the German translation of the Fear of Positive Evaluation Scale [70,71], consisting of 10 items asking to what degree participants experienced positive evaluations as anxiety-inducing (α=.81). Items are rated on a 10-point Likert scale (0=not at all true, 9=very true). Fear of negative evaluation was assessed using the revised Brief Fear of Negative Evaluation Scale [72] (German version: [73]). Participants rate 12 items on a 5-point Likert scale (1=not at all characteristic of me, 5=extremely characteristic of me). Cronbach α was 0.94.

#### Social Comparison

Social Comparison was evaluated with the Social Comparison Scale [74] (German translation [75]). This scale measures upward social comparison evaluations across 11 different comparison dimensions. Participants respond to bipolar items on a scale from 1 to 10 (eg, In relationship to others I feel:” 1=incompetent, 10=more competent). Higher scores reflect a more favorable self-assessment relative to others, while lower scores reflect a less favorable self-assessment (α=.87).

#### Trait Anxiety

Trait Anxiety was assessed using the Trait subscale of the State-Trait-Anxiety Scale [76,77]. Participants rate 10 items ranging from 1=not at all to 4=extremely (α=.81).

#### Stress

Stress was measured using the Perceived Stress Scale-10 [78]. The 10 items assess the degree of stress experienced in the past month (1=never, 5=very often). Cronbach α was 0.81.

#### Depression Level

Depression level was assessed using the Beck Depression Inventory-Fast Screen [79] (German version [80]). Participants respond to 7 items assessing various depressive symptoms (sadness, pessimism, past failure, loss of pleasure, self-dislike, self-criticalness, and suicidal ideation). The scores vary between 0 and 21, where higher scores signify more severe depressive symptoms (α=.75).

#### Impulsivity

Impulsivity was assessed using the shortened Barratt Impulsiveness Scale-15 [81] (German version [82]) consisting of 15 items (α=.76). This scale measures 3 subtraits (motor, attentional, and nonplanning) on a 5-point Likert scale (1=never, 4=always).

#### Empathizing

Empathizing was measured with the German translation of the Empathy Quotient-10 [83]. This questionnaire is associated with autistic traits and also exhibits a broad range within the general population [83]. The 10 items are scored on a 5-point Likert scale (1=strongly disagree, 4=strongly agree). Cronbach α was 0.70.

#### Neuroticism and Extraversion

Neuroticism and extraversion were assessed using the respective subscales of the Big Five Inventory [84] (German version [85]). Neuroticism and extraversion are highly predictive of depressive and anxiety disorders, as previous meta-analytic research has shown that individuals in various diagnostic groups exhibit high levels of neuroticism and low levels of extraversion [86,87]. Both subscales consist of 4 items scored by a 5-point Likert scale (1=strongly disagree, 5=strongly agree). Cronbach α for neuroticism was 0.74, and for extraversion, 0.82.

#### Self-Esteem

Self-esteem was assessed with the Rosenberg Self-Esteem Scale [88] (German version [89]). Participants rate 10 items on a 6-point Likert scale (1=not at all, 6=very much). Cronbach α was 0.90.

#### Subjective Social Status

Subjective social status was measured using the MacArthur Subjective Social Status Scale [90] (German version [91]). Participants rate their socioeconomic position (income, education, and occupation) relative to their community on a scale of 1 to 10, visualized as a ladder with 10 rungs.

#### Power

Power refers to one’s belief in having influence over others. It was assessed using the German translation of the Personal Sense of Power Scale [92,93], consisting of 6 items on a 7-point Likert scale (1=strongly disagree, 7=strongly agree). Cronbach α was 0.76.

#### Dominance and Prestige

Dominance and Prestige are 2 facets of the social rank attainment model, which posits that social hierarchy is achieved through force (dominance) or competence (prestige) [94]. These were assessed using the Dominance and Prestige Scale [95], which is a previously validated scale consisting of 2 subscales with 8 items measuring dominance (α=.77) and 9 items measuring prestige (α=.69), ranging from 1=not at all to 7=very much.

#### Agency

Agency was evaluated using the German adaptation of the Inventory of Interpersonal Problems-32 [96]. This tool comprises 32 items addressing 8 dimensions of interpersonal issues (α=.82), categorized into 2 overarching dimensions of agency and communion [97]. Participants indicate the extent to which each interpersonal challenge distresses them on a 5-point Likert scale (0=not at all, 4=extremely). For this study, the agency subscale score was used ([96]). This score specifically addresses interpersonal difficulties related to dominance, agency, and control [97].

Table 1 presents summaries of self-report measures. Responses in anxiety and depression measures showed that 27 (21.09%) participants exceeded the suggested diagnostic threshold of 34 for probable social anxiety [98] and 26 (20.31%) participants met the proposed cutoff score of 4 for mild depression [99].

**Table 1:** Descriptive statistics for self-report measures of participants completed before interaction, divided into measures for social anxiety, general psychopathology, and verticality (N=128).

Measure		Mean (SD; range)		Possible range^a^
**Social anxiety**				
	Social interaction anxiety	25.20 (11.58; 3 to 53)	0 to 80	
	Fear of negative evaluation	35.45 (10.16; 15 to 58)	12 to 60	
	Fear of positive evaluation	22.69 (12.21; 0 to 54)	0 to 90	
	Social comparison	65.79 (11.45; 40 to 97)	40 to 100	
**General psychopathology**				
	Trait anxiety	20.32 (4.36; 11 to 33.5)	10 to 40	
	Stress	27.24 (4.99; 18 to 42)	10 to 50	
	Depression levels	2.20 (2.32; 0 to 9.4)	0 to 21	
	Impulsivity	2.19 (0.38; 1.33 to 3.2)	1 to 4	
	Empathizing	12.21 (3.13; 3 to 19)	0 to 20	
	Neuroticism	3.20 (0.81; 1.75 to 5)	1 to 5	
	Extraversion	3.50 (0.84; 1.5 to 5)	1 to 5	
	Self-esteem	4.67 (0.77; 2.28 to 5.9)	1 to 6	
**Verticality**				
	Social status	5.88 (1.22; 3 to 10)	1 to 10	
	Power	29.52 (4.27; 17 to 38)	6 to 42	
	Dominance	2.59 (0.87; 1 to 5)	1 to 7	
	Prestige	4.91 (0.65; 3 to 6.33)	1 to 7	
	Agency	–0.79 (0.50; –1.93 to 0.66)	–2.90 to 2.90	

^a^Minimum and maximum scores that can be reached on each measure.

### Behavioral Measures

We examined several behavioral and physiological markers during the interaction. For eye gaze, we followed conventional thresholds [100,101] and excluded eye-tracking data recorded during blinks as well as 100 ms before and after each blink to avoid blink-related artifacts (average data loss 3.85%, SD 2.81% across participants). Missing data were interpolated using the Piecewise Cubic Hermite Interpolating Polynomial and smoothed with a second-order Butterworth filter (cutoff frequency=10 Hz). Gaze to the partner’s eye region was defined as visual attention directed toward the partner’s eye region, operationalized as within 2 degrees of visual angle around the center of a partner’s eye [102,103]. To account for common minor eyesight irregularities (eg, strabismus), gaze was coded to be directed toward a partner’s eyes if at least one of the participant’s eyes or a ray combining the 2 eyes was directed toward one of the partner’s eyes. Following previous research on differential gaze behavior during conversational roles [104], gaze was analyzed separately for speaking and listening phases.

Speaking behavior was defined as continuous time periods during which the sound amplitude recorded by the head-mounted display’s microphone exceeded a threshold of 2. Speech segments shorter than 1 second were classified as backchanneling vocalizations (eg, “ok” or “hmm”) and not counted as speaking turns, consistent with prior research on nonverbal behavior [105,106]. Speaking turns of one person could include brief pauses of up to 2 seconds before being classified as 2 consecutive turns from the same person. The threshold was established based on Heldner and Edlund [107], indicating that typical within-speaker pauses last around 1 second, and was specifically selected to avoid classifying brief hesitations as new turns or fusing separate turns. Furthermore, moments when 1 participant remained silent while their partner spoke were categorized as listening [108]. Instances of overlapping speech were classified as interruptions, attributed to the participant who began speaking while previously listening [109].

Smiling was measured using the head-mounted display’s built-in face tracking, which detects facial muscle activity based on action units defined by the Facial Action Coding System [110]. The intensity of smiling behavior was calculated by averaging the activation values from both sides of the mouth, yielding a score between 0 (no smile) and 100 (maximum smile expression).

Electrocardiograms were recorded using BItalino devices [111] at a sampling rate of 1000 Hz. QRS complexes (ventricular complex) were first detected automatically using an algorithm described by Elgendi et al [112]. Data were visually inspected individually and corrected manually if needed. Equidistantly sampled RR interval (duration between successive heartbeats) series were constructed using cubic spline interpolation. Heart rate (in beats per minute) was defined as 60 divided by the mean of the RR interval series (in seconds). To obtain HF-HRV, RR interval time series were first resampled at 4 Hz using bandlimited interpolation. Power spectral density was estimated using the Welch method, with 30-second Hann-windowed segments and 50% overlap. Each segment was processed using the Fast Fourier Transform, and only positive frequencies were retained. HF-HRV was quantified by integrating the power spectral density within the high-frequency band (0.15-0.4 Hz) and log-transforming the resulting power values.

As reported in Son and Rubo [41], participants spent an average of 23.05% (SD 11.96%) of their speaking time and 49.45% (SD 16.96%) of their listening time gazing toward their partner’s eyes. The mean proportion of speaking time was 50.73% (SD 9.90%). The mean proportion of time spent interrupting was 4.82% (SD 3.79%), and the average length of pauses before responding to the partner was 1.36 (SD 0.70) seconds. Average speech loudness was 2.70 (SD 0.77), measured relative to the maximum microphone recording capacity (100). The average smiling magnitude was 13.40 (SD 7.99) over the entire conversation period. The mean heart rate was 79.61 (SD 11.95) beats per minute, and the average HF-HRV was 6.36 (SD 0.96).

### Data Analysis

Analyses were performed using R software (version 4.3.2; R Foundation for Statistical Computing) [113]. In the first step, principal component analysis was applied to self-report measures related to social anxiety, general psychopathology, and verticality (dominance and power), respectively. Principal component analysis is a widely used method to derive a common component from variables, which maximizes the total variance [60]. Focusing on the dominant components captures the most significant sources of variability in the data, providing a parsimonious representation of the underlying constructs and helping to avoid overly specific interpretations [23].

To further validate these components, two-sided Pearson correlations were conducted between the extracted components and the individual self-report measures that contributed to each factor. For these analyses, measures were aggregated across the entire conversation period. As expected, measures of social anxiety, psychopathology, and verticality were each correlated with their respective common factors (Multimedia Appendix 1). Consequently, subsequent analyses were carried out using these first dominant factors. The effect of each factor on behavioral and physiological measures was examined using mixed-effects models, with random intercepts for participant ID as well as random slopes for topics nested within ID to account for within-subject variability. For all variables, values beyond 3 SD from the mean were set to 3 SD from the mean and *z*-standardized prior to analyses. The percentage of corrected outliers ranged from 0.78% to 3.13% (Multimedia Appendix 1). The significance of results was determined with α set to 5%. *P* values were adjusted for multiple comparisons using the Benjamini-Hochberg false discovery rate (FDR) procedure.

To reduce potential bias, analyses were preregistered, data acquisition was standardized, and preprocessing algorithms were held identical for all participants. Furthermore, mixed-effects models were used to account for within-subject and sampling variability.

## Results

### Effect of Psychological Traits on Behavior

**Figure 3:**
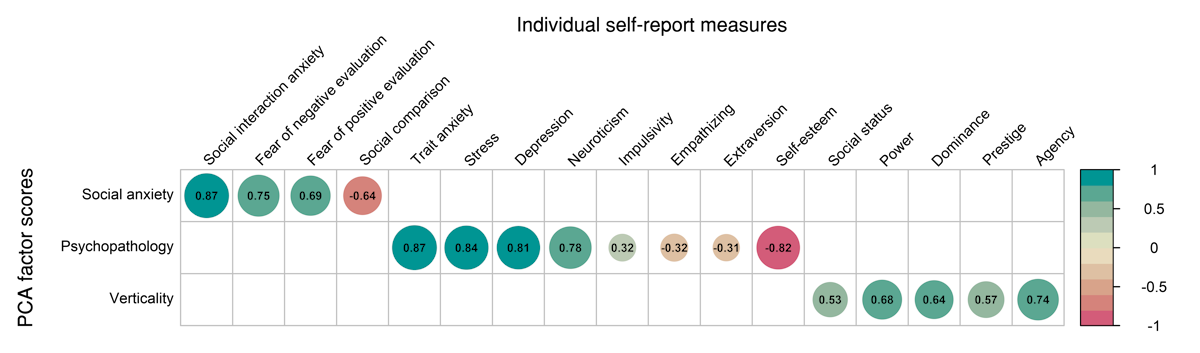
Pearson correlation coefficients between each first component of the principal component analysis (PCA) for social anxiety, general psychopathology, and verticality with self-report measures on individual scales. As expected, results show overall strong convergence between self-report measures relating to each of the 3 overarching trait constructs.

Principal component analyses were conducted separately for self-report measures related to social anxiety, general psychopathology, and verticality, which reflects dominance and rank. Each analysis yielded a dominant first component (Multimedia Appendix 1). All factors showed theoretically consistent and large associations with their corresponding measures, confirming their suitability as parsimonious representations of the underlying traits (Figure 3). Detailed results are provided in Multimedia Appendix 1.

**Figure 4:**
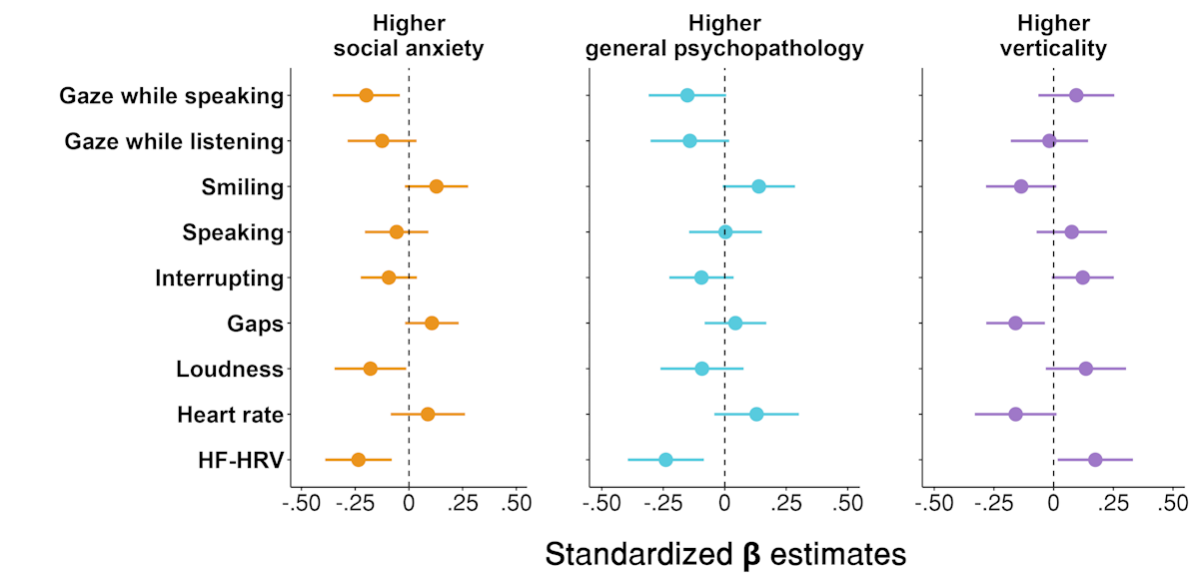
Associations between social anxiety, general psychopathology, and verticality with behavioral and physiological measures collected during the social virtual reality interaction. Values are standardized β estimates, indicating lower or higher measurements on behavioral or physiological metrics with higher trait expression. For example, higher social anxiety predicts reduced gaze to the partner’s eyes while speaking. Lines represent 95% CIs. HF-HRV: high-frequency heart rate variability.

The effect of social anxiety, general psychopathology, and verticality on the assessed behavioral measures was examined in linear mixed models. Figure 4 shows the *β* estimates and CIs for the relationships between factors and behaviors. A detailed summary of results is shown in Multimedia Appendix 1.

Higher social anxiety levels were associated with reduced gaze toward the partner’s eyes during one’s own speaking turn (*β*=–.20, 95% CI –0.35 to –0.04; t_126_=–2.51; *P*=.01). The effect of underlying psychopathology on the amount of gaze toward the partner’s eyes while speaking (*β*=–.15, 95% CI –0.31 to 0.01; t_126_=–1.90; *P*=.06) and while listening was marginally significant (*β*=–.14, 95% CI –0.30 to 0.02; t_126_=–1.75; *P*=.08). The association between social anxiety and psychopathology with smiling behavior was marginally significant, such that higher social anxiety (*β*=.13, 95% CI –0.02 to 0.28; t_126_=1.72; *P*=.09) and higher psychopathology (*β*=.14, 95% CI –0.01 to 0.29; t_126_=1.87; *P*=.06) was positively associated with smiling. In contrast, verticality was marginally significantly associated with less smiling behavior (*β*=–.14, 95% CI –0.28 to 0.01; t_126_=–1.83; *P*=.07).

Social anxiety was furthermore associated with reduced speaking volume (*β*=–.18, 95% CI –0.35 to –0.01; t_126_=–2.12; *P*=.04). The effect of social anxiety on gaps was marginally significant, such that higher social anxiety was associated with longer gaps between one’s speaking turns (*β*=.11, 95% CI –0.02 to 0.23; t_126_=1.70; *P*=.09). Verticality influenced gap duration, such that higher verticality was associated with shorter gaps before responding (*β*=–.16, 95% CI –0.28 to –0.04; t_126_=–2.57; *P*=.01). Verticality was additionally marginally significantly associated with interrupting behavior, such that higher verticality was associated with more interrupting behavior (*β*=.12, 95% CI –0.03 to 0.25; t_126_=1.86; *P*=.07). The effect of verticality on heart rate was marginally significant, such that individuals high in verticality displayed lower heart rate (*β*=–.16, 95% CI –0.33 to 0.01; t_119_=–1.84; *P*=.07).

Social anxiety and psychopathology were associated with reduced HF-HRV (social anxiety: *β*=–.23, 95% CI –0.39 to –0.08; t_119_=–3.00; *P*=.003; psychopathology: *β*=–.24, 95% CI –0.39 to –0.08; t_119_=–3.07; *P*=.003), whereas higher verticality was associated with increased HF-HRV (*β*=.17, 95% CI 0.02 to 0.33; t_119_=2.20; *P*=.03). After adjusting for multiple comparisons, the observed significant effects only held for social anxiety and psychopathology on HF-HRV.

### Behavioral Similarities Between Social Anxiety, Psychopathology, and Verticality

We additionally compared similarities and differences in how social anxiety, psychopathology, and verticality related to behavior. Pearson correlations on the obtained *β* estimates were performed to examine the pattern of behavior between the 3 factors. The analysis revealed very strong correlations between the factors, such that the pattern of behavior in social anxiety was strongly positively associated with that in psychopathology (*r*=0.94, 95% CI 0.75 to 0.99; *t*_7_=7.61; *P*<.001), and the pattern of behavior in verticality was highly negatively associated to that in social anxiety (*r*=–0.92, 95% CI –0.98 to –0.65; *t*_7_=–6.14; *P*<.001) and psychopathology (*r*=–0.83, 95% CI –0.96 to –0.38; *t*_7_=–3.98; *P*=.005).

## Discussion

### Principal Results

We examined whether previously reported associations between social anxiety and behavioral and physiological markers could be traced during real-time, dyadic interaction within social VR. This platform enables the automated assessment of fine-grained behavioral data during lifelike dyadic interactions, which has remained relatively difficult to assess in previous paradigms. We found that behavioral and physiological patterns previously associated with social anxiety also emerged in interactions in social VR, replicating patterns observed in face-to-face interactions. These associations were not exclusive to social anxiety but were also found in relation to broader psychopathology and lowered verticality.

Findings suggested both shared and distinct behavioral patterns across psychological traits. Gaze avoidance was associated with social anxiety, replicating previous findings from face-to-face interaction studies [4,6] and from social VR studies involving agent-based interactions [114]. This effect was specific to speaking phases, consistent with prior work showing heightened gaze avoidance during speech, but not during listening [7,8]. While some studies have reported stronger associations with social anxiety during listening or found no clear phase-specific effects [9,10], these results support the view that gaze behavior in social anxiety may be particularly sensitive to moments of self-expression, when perceived social evaluation is likely heightened. In addition, reduced loudness was relatively specific to social anxiety, aligning with earlier research linking reduced loudness of speech to heightened nervousness in patients with social anxiety [15].

Although associations between social anxiety and behavior more consistently reached statistical significance compared to general psychopathology, the overall pattern of results was highly similar. Both gaze behavior during speaking and listening, as well as smiling, showed trends in the expected direction with higher general psychopathology. These findings suggest that certain interaction behaviors may also reflect broader psychological vulnerability in interpersonal settings. Furthermore, these findings may also reflect the heterogeneity of general psychopathology, which spans both internalizing and externalizing symptoms and may therefore give rise to less distinct patterns of social behavior. Future experimental studies that systematically manipulate the social-evaluative context could help determine whether gaze avoidance is a specific behavioral marker for social anxiety, or whether it generalizes to other dimensions of psychopathology [23].

Conversely, verticality, which encompasses constructs such as dominance and status, showed a partly complementary behavioral profile, marked by increased gaze during speaking (but not listening) and shorter turn-taking gaps—patterns previously linked to assertive or high-dominance behavior [31,32]. These results align with social rank theory [29], which posits that perceived social status regulates behavior in interpersonal contexts. Individuals higher in verticality may perceive less social threat and engage more confidently, reducing the need for avoidant or submissive strategies.

While these associations were theoretically coherent, most were small and did not remain significant after adjusting for multiple comparisons. Nonetheless, the pattern of results was consistent across measures and aligned with theoretical expectations. For instance, 6 of 36 bivariate associations were at least significant at *P*<.05 (expected by chance: 1.8), and 14 were at least marginally significant at *P*<.10 (expected by chance: 3.6). Importantly, all of these associations were in the expected direction, suggesting that the overall pattern aligns with hypothesized relationships.

Regarding physiological data, HF-HRV was predictive for all 3 traits, decreasing with higher social anxiety and general psychopathology, and increasing with verticality. As a physiological index of autonomic regulation and stress, reduced HF-HRV is broadly linked to impaired emotion regulation and social withdrawal [17,19,20,115]. In contrast to behavioral markers, this pattern suggests that HF-HRV may capture a general dimension of stress sensitivity, consistent with its proposed role as a transdiagnostic marker of stress [116,117].

Taken together, behavioral modulations in social anxiety and psychopathology were highly parallel, with verticality showing a largely inverted pattern. Both social anxiety and broader psychopathology were overall associated with an increase in interpersonal safety behaviors, particularly those expressed through submissive strategies such as reduced assertiveness and appeasing social cues [4,118]. These patterns are consistent with prior work linking these traits to diminished perceptions of social power [25,27]. Submissive behavior, in turn, has been consistently linked to reduced psychological well-being [119], reinforcing its clinical relevance as an important marker for heightened psychological vulnerability [27]. Conversely, verticality was associated with reduced submissive behavior, suggesting it may represent the opposing pole of a social-behavioral continuum relative to the submissive tendencies observed in social anxiety and broader psychopathology.

In addition, the strong behavioral convergence between social anxiety and general psychopathology lends support to the *p*-factor framework as a useful model for understanding shared variance in social behavior across psychological traits [23,24]. The behavioral overlap between social anxiety and general psychopathology indicates that the specificity of commonly used markers warrants further examination: rather than uniquely reflecting any single construct, interaction behavior may commonly indicate broader impairments in social functioning and well-being [120,121].

### Implications

Our results highlight important implications. Compared to traditional eye-tracking paradigms [35] or smartphone phenotyping [38], our study demonstrates that social VR enables the synchronous collection of behavior during real-time interactions, enabling the study of social dynamics and mutual feedback that characterize human interactions. In social VR, participants can express and observe natural interaction behavior as it unfolds in a shared environment, creating conditions for more ecologically valid expressions of gaze, speech, and physiological responses [36]. This highlights this medium’s potential as a powerful research tool for studying social processes and understanding psychological traits in socially embedded contexts.

Social VR presents promising opportunities for clinical research and novel forms of mental health care. Recent findings show that it is not only a growing digital medium, especially among younger users, but also a psychologically meaningful space for interactions. Supportive interactions in these environments have been linked to greater social presence, enhanced self-efficacy, and reduced loneliness [122]. As such, the technique may offer a valuable context for large-scale, ecologically valid monitoring of social behavior among multiple users.

Social VR also offers distinct advantages as a medium for delivering remote mental health care. Unlike conventional videoconferencing, it preserves key nonverbal cues such as eye contact and conversational timing, helping to overcome limitations such as reduced social presence and Zoom fatigue [123]. Moreover, it may enable novel therapeutic formats that are difficult to implement in traditional settings, such as fully anonymous group therapy sessions, which may lower barriers for individuals with social anxiety or those who fear stigmatization.

In addition, its capacity for automated data capture allows researchers to conduct passive, high-frequency assessments, which may be extended toward repeated interactions—such as multiple psychotherapy sessions or ongoing team meetings in virtual environments—facilitating the continuous monitoring of changes in well-being. Similar to passive smartphone phenotyping, which has demonstrated that passive sensor data can predict symptom changes in mental disorders [124], social VR offers new opportunities to assess social functioning in ecologically valid, interactive contexts. For example, fine-grained behavioral data collected in VR may aid in early risk detection, provide objective measures of therapeutic progress, or support the tailoring of interventions to individual behavioral patterns. Such data may be used to inform clinical decisions or even provide feedback within the VR setting itself [125].

At the same time, the technique’s capability for unobtrusive, fine-grained data collection may also introduce significant security and ethical challenges. The passive and automated nature of data collection in these environments, which can be carried out by a server as well as any other computer in the network simulation, increases the risk of sensitive behavioral data being collected without the user’s knowledge. While data collection in this study was conducted using an in-house software tool with no reliance on external services or internet connection, large-scale implementations of social VR in research contexts and beyond will be required to carefully consider data security principles and ethical implications of their use [39,126].

### Limitations

We mapped a range of behavioral and physiological markers to interindividual differences in self-reported psychological traits in a naturalistic social VR interaction in 128 participants. Despite their strengths, 6 factors limit the generalizability of the findings.

First, the sample was composed of students from the German-speaking region of Switzerland, which may have introduced selection bias and limited the representativeness of the findings. Previous research indicates that social behaviors can vary across the lifespan; for example, avoidant behaviors such as gaze aversion tend to increase during adolescence [127]. Cultural factors may likewise influence nonverbal behavior and have been linked to differences in satisfaction and even health outcomes in cross-cultural clinical contexts [128]. As many aspects of nonverbal communication are culturally learned, the generalizability of our findings may decrease with greater cultural distance from the studied population [129]. At the same time, it was observed that interindividual differences in traits such as social anxiety often modulate modes of experience and behavior across population subgroups [130].

Second, although the sample showed substantial variability across behavioral and trait measures—with approximately 1 in 5 participants meeting thresholds for social anxiety disorder—the study lacked sufficient statistical power to examine gender differences in behavior, as the sample consisted predominantly of female participants. Exploratory analyses from a separate report [41] indicated that women smiled more frequently than men, consistent with prior findings in nonverbal behavior research [131]. However, the unequal group sizes in this study limit the interpretability of gender effects. Future research should therefore aim to systematically examine how demographic and cultural factors moderate behavioral dynamics in VR interactions to achieve more generalizability.

Third, the prevalence of mental disorders among university students is comparable to that in the general population [132], and psychological traits examined here—such as general psychopathology—are increasingly understood as dimensional rather than categorical [133]. Although some participants exceeded clinical thresholds for anxiety or depression, a clinically diagnosed sample was not included. It remains uncertain whether the behavioral modulations observed in this nonclinical sample would extend to full-blown mental disorders. In particular, stronger expressions of psychopathology may be associated with more specific modulations of interaction behavior than those observed here [134]. However, prior research has also shown that subclinical social anxiety typically exhibits behavioral patterns similar to those in social anxiety disorder, such as heightened anxiety in facial expressions [135]. Likewise, physiological indices such as heart rate have been shown to distinguish subclinical social anxiety during speech tasks in young adults [136]. These findings support the notion of social anxiety as a continuum, suggesting that the patterns observed here may represent expressions along the same underlying spectrum.

Fourth, while we included a broad range of self-report measures covering internalizing symptoms (eg, anxiety or depression) and select externalizing symptoms (eg, impulsivity or empathizing), other common externalizing symptoms such as substance abuse and aggressive behaviors were not assessed, as these may be less commonly observed in a student sample. This omission may have limited the scope of the general psychopathology construct captured. However, internalizing and externalizing psychopathology have been shown to be highly correlated across various age groups [137,138], and prior research has successfully modeled general psychopathology without including externalizing dimensions [139]. Nonetheless, future research should incorporate a broader range of symptom domains to more comprehensively assess the influence of psychopathology on social behavior.

Fifth, the study adopted a correlational design without a comparison condition in which participants interacted outside of VR. It therefore remains uncertain whether absolute behavioral levels in VR fully align with those observed in real-world interactions. A direct in vivo-in virtuo comparison would provide a stronger test of behavioral equivalence. However, our previous work using the same dataset [41] compared key interaction metrics to established face-to-face benchmarks and found highly similar patterns, consistent with other recent findings comparing VR to real-life interactions [40], which suggests that fundamental aspects of social behavior are preserved in this social VR setting.

Lastly, although the presence of cohesion between stable traits and observed behavior implies some level of stability in behavior, our design did not allow for directly testing within-person consistency across interactions with varying partners. In order to better characterize how interpersonal processes contribute to individual variation in behavior [140], future studies should assess behavior in individuals across various interactions.

### Conclusion

We investigated behavioral and physiological correlates of social anxiety as well as broader psychopathology and verticality in dyadic interactions in social VR. Extending previous work that examined interpersonal dynamics in face-to-face settings or responses to virtual agents, this study provides a comprehensive account of behavioral and physiological modulations associated with social anxiety in real-time avatar-based human-human interactions. In an integrated analytical framework, we assessed gaze, speaking, smiling expression, as well as heart rate and HF-HRV, and their associations with individual traits. As observed in natural face-to-face interactions, social anxiety levels were associated with behavioral modulations such as quieter speaking and reduced eye gaze to a partner while speaking and with reduced HF-HRV, indicating increased social stress and submissiveness. Behavioral and physiological patterns were not exclusive to social anxiety but appeared to reflect broader psychological characteristics, including higher general psychopathology and lower verticality. These results indicate that social interaction patterns may reflect general psychological vulnerability rather than effects specific to social anxiety levels. Behavioral traces that can be obtained automatically in social VR carry sensitive psychological information and, problematically, may be used for unintended profiling or psychological inference. At the same time, these data also provide valuable opportunities to assess psychologically relevant behavioral patterns in social interactions at scale in various settings, ultimately helping to understand pathological mechanisms in social interaction behavior and improving psychotherapy.

## Appendix

Multimedia Appendix 1 Results with detailed explanations.

## Abbreviations

HF-HRV: high-frequency heart rate variability
SIAS: Social Interaction Anxiety Scale
STROBE: Strengthening the Reporting of Observational Studies in Epidemiology
VR: virtual reality

## Data Availability

This study’s sample size, measures, and analysis approach were preregistered [54]. The study conformed to the principles expressed in the Declaration of Helsinki and was approved by the local ethics committee at the University of Bern (reference number 2023-09-01). Data that support the findings of this study are available at [53].
