# Supplementary material for: Automated Inference of Social Anxiety From Behavior in Social Virtual Reality: Cross-Sectional Observational Study
Source: J Med Internet Res. 2026 Mar 11;28:e79147. doi: 10.2196/79147 (PMC13019030; doi:10.2196/79147)
Supplement: Multimedia Appendix 1 [file jmir_v28i1e79147_app1.docx]

# Supplementary to: Automated Inference of Social Anxiety from Behavior in Social Virtual Reality

Gayoung Son${}^{1}$, M. Sc.; Marius Rubo${}^{1}$, PhD

${}^{1}$Cognitive Psychology, Perception and Research Methods, Institute of Psychology, University of Bern, Switzerland

### Outliers

To calculate outliers, the data were averaged across all conversation phases for each participant. Data points identified as outliers, exceeding 3 $SD$ from the mean, were adjusted to exactly 3 $SD$ from the mean. For self-reported measures, outliers were found in three different measures. One value was corrected in trait anxiety (STAI: 0.78%, 3.11 $SD$ above the mean), four values in depression levels (BDI-FS: 3.13%, 3.25, 3.68, 3.25, 3.25 $SD$ above the mean) and one value in self-esteem (SES: 0.78%, 3.11 $SD$ below the mean). The proportion of modified outlier values in the behavioral measures spanned from 0% to 2.08% (refer to Table S1).

Table S1: Percentage of Corrected Outliers of Behavioral Variables

| **Variable** | **Percentage Outliers (%)** |
| --- | --- |
| Gaze While Speaking | 1.04 |
| Gaze While Listening | 0 |
| Gaze Beginning of Turn | 1.56 |
| Gaze End of Turn | 0.26 |
| Smile | 0.52 |
| Percent Speaking | 0.78 |
| Percent Interrupting | 2.08 |
| Turn duration | 0.78 |
| Gaps | 0.53 |
| Loudness | 0.78 |

### Exclusions and Missing Data

A total of 16 participants were excluded prior to reaching the preregistered sample size due to data quality issues. Specifically, seven dyads were excluded because facial expressions failed to record, one dyad because the computer connection was lost, and one dyad because they reported speaking English. In accordance with the preregistered protocol, these participants were replaced to ensure a complete dataset. A small proportion of data were missing due to predefined criteria for speaking behavior, such as constant interruptions during responses (n = 3, 2.34%). In addition, heart rate data from seven participants (5.47%) were excluded due to poor-quality interbeat interval (IBI) signals caused by artifacts or interruptions in recording. Little’s Test [1] indicated that these missing data were completely random (P > .05).

### Conversation Topics

In the study, participants conversed for a total of 30 minutes on three different topics. Detailed implementations are reported in [2]. For small talk, participants engaged in discussions centered on three everyday topics, such as the weather or their mode of travel to the examination. An example question is: "How has today been for you so far?".

For personal talk, participants engaged in discussions designed to promote closeness on deeper, personal topics. Example questions included: "What does friendship mean for you?". For opinion talk, participants discussed controversial topics on which their viewpoints differed, and were instructed to convince the other of their own opinion. An example question is: "Should cannabis be legal?".

Small talk and personal talk questions were taken from [3], while opinion talk questions were selected from a pool of questions from a pilot survey. To maximize the likelihood of participants holding opposing viewpoints, we selected the three questions with the highest variance in response distributions from the survey. This approach was based on findings that perceived differences in opinions on controversial subjects can contribute to distress [4].

### Avatar Creation

Participant pairs interacted exclusively within the Social VR environment, each represented by a virtual avatar. These avatars mirrored participants’ head and eye movements, blinking, mouth movement, facial expressions, and gestures in real time. Avatars were created using Character Creator 4 [5] and the Headshot plugin, which generates realistic 3D models from a single portrait photo. For each avatar, facial features such as eye color, blendshapes, hair, and body size were manually adjusted by a research assistant. All avatars wore identical clothing, and only the head, torso, and hand silhouettes were visible in the VR environment.

In the self-similar condition, avatars were generated to resemble the participants based on their portrait. In the generic condition, twelve male and female avatars were created from artificially generated portraits of non-existing individuals [6], with gender matched and assigned randomly. Avatars were exported as FBX files with the CC4 extended expression profile and 15 visemes (1:1 Direct system: [5]). The expression profile includes 140 blendshapes for detailed facial expression control. Avatars were deployed on both computers using a dedicated software tool [7].

## Principal Component Analyses

Principal component analysis was applied to self-report measures related to social anxiety, general psychopathology and verticality, respectively. PCA is a widely-used method to derive a common component from variables, which maximizes the total variance [8]. By focusing on these dominant components, we capture the most significant sources of variability in the data, providing a parsimonious explanation of the underlying constructs. Consequently, subsequent analyses were carried out using these first strongest components. Correlations between these factors and individual self-report measures were analysed using Pearson’s correlations, where behavioral measures are aggregated across the entire conversation period.

Results indicated dominant first factors for each three constructs, each explaining a significant proportion of variance across the respective measures (see Figures [1](#fig:PCA_SA) - [3](#fig:PCA_V)). For social anxiety, the first component exhibited an eigenvalue larger than 1 (2.20), explaining 55.10% of variance. For psychopathology, the first three components showed an eigenvalue larger than 1 (3.70, 1.20 and 1.08). The first component accounted for the largest contribution, explaining 46.30% of the total variance. For verticality, two components had eigenvalues larger than 1 (2.03 and 1.12), with the first component accounting for 40.54% of the variance.

|  |
| --- |

Figure S1: Results from principal component analysis (PCA) applied to measures related to social anxiety. (a) presents the eigenvalues of the factors’ as a scree plot, and (b) illustrates the correlations between the initial three factors and individual variables. The substantial first factor was interpreted as indicating social anxiety.
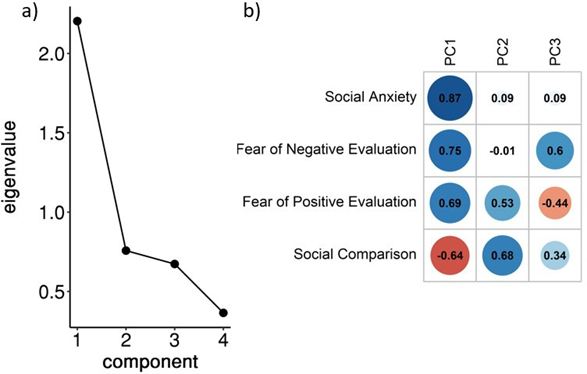


|  |
| --- |

Figure S2: Results from principal component analysis (PCA) applied to measures related to general psychopathology. (a) presents the eigenvalues of the factors’ as a scree plot, and (b) illustrates the correlations between the initial three factors and individual variables. The substantial first factor was interpreted as indicating general psychopathology.
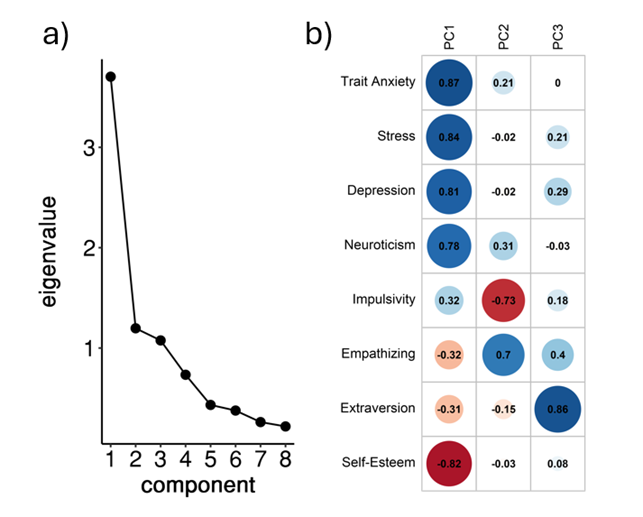


|  |
| --- |

Figure S3: Results from principal component analysis (PCA) applied to measures related to verticality. (a) presents the eigenvalues of the factors’ as a scree plot, and (b) illustrates the correlations between the initial two factors and individual variables. The first factor was interpreted as indicating verticality.
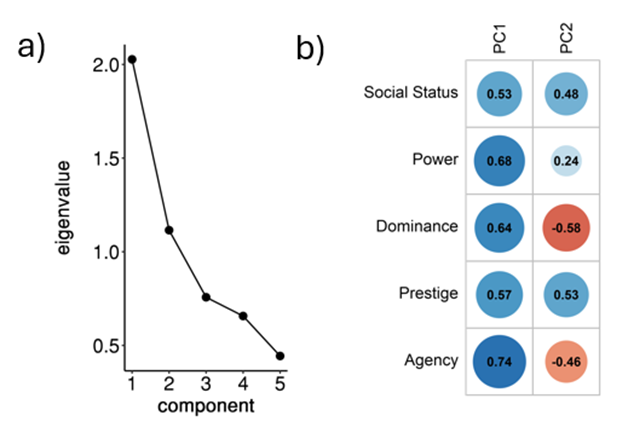


#### Correlations With Self-Report Measures

We calculated Pearson’s correlations between each first factor and the related self-report measures to evaluate the strength of their associations and to validate the components as representations of the underlying constructs. All initial factors aligned with theoretical expectations and demonstrated the hypothesized associations with self-reported measures, supporting their robustness as representations of the constructs.

The strongest first factor for social anxiety showed positive and large correlations with social interaction anxiety ($r$ = 0.87, 95% CI [0.82, 0.90], $t$(126) = 19.55, $p$ $<.$001), fear of negative evaluation ($r$ = 0.75, 95% CI [0.67, 0.82], $t$(126) = 12.83, $p$ $<.$001), fear of positive evaluation ($r$ = 0.69, 95% CI [0.59, 0.77], $t$(126) = 10.75, $p$ $<.$001) and a negative and large correlation with positive social comparison tendencies ($r$ = -0.64, 95% CI [-0.73, -0.52], $t$(126) = -9.30, $p$ $<.$001).

The strongest first factor for general psychopathology showed positive and very large correlations with trait anxiety ($r$ = 0.87, 95% CI [0.82, 0.91], $t$(126) = 19.88, $p$ $<.$001), stress ($r$ = 0.84, 95% CI [0.78, 0.89], $t$(126) = 17.49, $p$ $<.$001), depressive levels ($r$ = 0.81, 95% CI [0.75, 0.87], $t$(126) = 15.73, $p$ $<.$001), neuroticism ($r$ = 0.78, 95% CI [0.70, 0.84], $t$(126) = 13.82, $p$ $<.$001). Impulsivity showed a large and positive correlation ($r$ = 0.32, 95% CI [0.15, 0.47], $t$(126) = 3.76, $p$ $<.$001). Large and negative correlations were observed between the first factor and empathizing ($r$ = -0.32, 95% CI [-0.47, -0.16], $t$(126) = -3.85, $p$ $<.$001) and extraversion ($r$ = -0.31, 95% CI [-0.46, -0.14], $t$(126) = -3.65, $p$ $<.$001). The correlation between the first factor and self-esteem was very large ($r$ = -0.82, 95% CI [-0.87, -0.75], $t$(126) = -16.00, $p$ $<.$001).

The strongest first factor for verticality exhibited very large and positive correlations with subjective ratings of social status ($r$ = 0.53, 95% CI [0.39, 0.64], $t$(126) = 6.95, $p$ $<.$001), power ($r$ = 0.68, 95% CI [0.57, 0.76], $t$(126) = 10.38, $p$ $<.$001), dominance ($r$ = 0.64, 95% CI [0.53, 0.73], $t$(126) = 9.44, $p$ $<.$001), prestige ($r$ = 0.57, 95% CI [0.44, 0.68], $t$(126) = 7.83, $p$ $<.$001) and agency ($r$ = 0.74, 95% CI [0.65, 0.81], $t$(126) = 12.37, $p$ $<.$001).

### Effect of Psychological Traits on Behavior

Table S2: Effects of Social Anxiety, Psychopathology, and Verticality on Behavior

| **Variable** | **Estimate** | **95% CI** | $t$**(df)** | $P$ | $P$**adj** |
| --- | --- | --- | --- | --- | --- |
| **Social Anxiety** | | | | | |
| Gaze While Speaking | -0.20 | [-0.35, -0.04] | -2.51 (126) | .013 | .058 |
| Gaze While Listening | -0.12 | [-0.29, 0.04] | -1.54 (126) | .127 | .190 |
| Smiling | 0.13 | [-0.02, 0.28] | 1.72 (126) | .087 | .165 |
| Speaking | -0.06 | [-0.20, 0.09] | -0.77 (126) | .445 | .445 |
| Interrupting | -0.09 | [-0.22, 0.04] | -1.41 (126) | .160 | .206 |
| Gaps | 0.11 | [-0.02, 0.23] | 1.70 (126) | .092 | .166 |
| Loudness | -0.18 | [-0.35, -0.01] | -2.12 (126) | .036 | .108 |
| Heart Rate | 0.09 | [-0.08, 0.26] | 1.01 (119) | .314 | .353 |
| HF-HRV | -0.23 | [-0.39, -0.08] | -3.00 (119) | .003 | .027 |
| **Psychopathology** | | | | | |
| Gaze While Speaking | -0.15 | [-0.31, -0.01] | -1.90 (126) | .059 | .184 |
| Gaze While Listening | -0.14 | [-0.30, 0.02] | -1.75 (126) | .082 | .184 |
| Smiling | 0.14 | [ 0.01, 0.29] | 1.87 (126) | .064 | .184 |
| Speaking | -0.00 | [-0.14, 0.15] | -0.05 (126) | .964 | .964 |
| Interrupting | -0.09 | [-0.23, 0.04] | -1.43 (126) | .155 | .232 |
| Gaps | 0.04 | [-0.08, 0.17] | 0.69 (126) | .491 | .552 |
| Loudness | -0.09 | [-0.26, 0.08] | -1.08 (126) | .282 | .363 |
| Heart Rate | 0.13 | [-0.04, 0.30] | 1.49 (119) | .138 | .232 |
| HF-HRV | -0.24 | [-0.39, -0.08] | -3.07 (119) | .003 | .027 |
| **Verticality** | | | | | |
| Gaze While Speaking | 0.10 | [-0.06, 0.25] | 1.18 (126) | .239 | .307 |
| Gaze While Listening | -0.02 | [-0.18, 0.14] | -0.21 (126) | .831 | .831 |
| Smiling | -0.14 | [-0.28, 0.01] | -1.83 (126) | .070 | .126 |
| Speaking | 0.08 | [-0.07, 0.22] | 1.02 (126) | .309 | .348 |
| Interrupting | 0.12 | [-0.01, 0.25] | 1.86 (126) | .065 | .126 |
| Gaps | -0.16 | [-0.28, -0.04] | -2.57 (126) | .011 | .099 |
| Loudness | 0.14 | [-0.03, 0.30] | 1.59 (126) | .114 | .171 |
| Heart Rate | -0.16 | [-0.33, 0.01] | -1.84 (119) | .069 | .126 |
| HF-HRV | 0.17 | [ 0.02, 0.33] | 2.20 (119) | .030 | .126 |

*Note.*: HF-HRV = High-frequency heart-rate variability. Estimates are standardized beta coefficients. Padj = p-values were adjusted within each factor using the Benjamini & Hochberg procedure [9].

### References

1. Little RJA. A test of missing completely at random for multivariate data with missing values. *Journal of the American Statistical Association*. 1988;83(404):1198-1202. doi:[10.1080/01621459.1988.10478722](https://doi.org/10.1080/01621459.1988.10478722)
2. Son G, Rubo M. Social virtual reality elicits natural interaction behavior with self-similar and generic avatars. *International Journal of Human-Computer Studies*. 2025;199:103488. doi:[10.1016/j.ijhcs.2025.103488](https://doi.org/10.1016/j.ijhcs.2025.103488)
3. Aron A, Melinat E, Aron EN, Vallone RD, Bator RJ. The experimental generation of interpersonal closeness: A procedure and some preliminary findings. *Personality and Social Psychology Bulletin*. 1997;23:363-377. doi:[10.1177/0146167297234003](https://doi.org/10.1177/0146167297234003)
4. Matz DC, Wood W. Cognitive dissonance in groups: The consequences of disagreement. *Journal of Personality and Social Psychology*. 2005;88:22-37. doi:[10.1037/0022-3514.88.1.22](https://doi.org/10.1037/0022-3514.88.1.22)
5. Reallusion. Reallusion. Published online.
6. Karras T, Laine S, Aila T. A style-based generator architecture for generative adversarial networks. *IEEE Transactions on Pattern Analysis and Machine Intelligence*. 2018;43:4217-4228. doi:[10.1109/TPAMI.2020.2970919](https://doi.org/10.1109/TPAMI.2020.2970919)
7. Rubo M. AvatarTransferPro: A tool for automatic transfer of realistic personalized avatars in networked social virtual reality. *SoftwareX*. 2024;27:101873. doi:[10.1016/j.softx.2024.101873](https://doi.org/10.1016/j.softx.2024.101873)
8. Schreiber JB. Issues and recommendations for exploratory factor analysis and principal component analysis. *Research in Social and Administrative Pharmacy*. 2021;17(5):1004-1011. doi:[10.1016/j.sapharm.2020.07.027](https://doi.org/10.1016/j.sapharm.2020.07.027)
9. Benjamini Y, Hochberg Y. Controlling the false discovery rate: A practical and powerful approach to multiple testing. *Journal of the Royal Statistical Society Series B: Statistical Methodology*. 1995;57:289-300. doi:[10.1111/j.2517-6161.1995.tb02031.x](https://doi.org/10.1111/j.2517-6161.1995.tb02031.x)
